# Effective CO_2_ capture by using poly (acrylonitrile) nanofibers based on the radiation grafting procedure in fixed-bed adsorption column

**DOI:** 10.1038/s41598-023-33036-y

**Published:** 2023-04-15

**Authors:** Ali Ahmadizadeh Tourzani, Faramarz Hormozi, Mehdi Asadollahzadeh, Rezvan Torkaman

**Affiliations:** 1 Faculty of Chemical, Gas and Petroleum Engineering, Semnan University, P.O. Box 35195-363, Semnan, Islamic Republic of Iran; 2 Nuclear Fuel Cycle Research School, Nuclear Science and Technology Research Institute, P.O. Box 11365-8486, Tehran, Islamic Republic of Iran

**Keywords:** Chemical engineering, Nanoscience and technology

## Abstract

In this study, a new adsorbent was investigated for CO_2_ adsorption in the fixed-bed column. Poly (acrylonitrile) nanofibers were prepared by electrospinning, then grafting under gamma irradiation with glycidyl methacrylate (GMA). Then, the nanofibers were modified with ethanolamine (EA), diethylamine (DEA) and triethylamine (TEA) to adsorb carbon dioxide molecules. Dynamic adsorption experiments were performed with a mixture of CH_4_, CO_2_ in a constant bed column at ambient pressure and temperature and CO_2_ feed concentration (5%). The maximum adsorption capacity is 2.84 mmol/g for samples with 172.26% degree of grafting (DG) in 10 kGy. Also, the degree of amination with ethanolamine was achieved equal to 170.83%. In addition, the reduction of the regeneration temperature and the stability of this adsorbent after four cycles indicated the high performance of this adsorbent for CO_2_ adsorption.

## Introduction

Global warming is now the most serious environmental and public health hazard^1^. Anthropogenic CO_2_ emissions from fossil fuel burning are the primary cause of global warming^2^. The intergovernmental panel on climate change (IPCC) is a non-profit organization (IPCC). Between 1970 and 2004, total human emissions, including methane, carbon dioxide, and nitrogen oxides, rose by 70% owing to human activity. Towards the year 2100, the IPCC predicts that carbon dioxide concentrations will reach 570 parts per million (ppm)^3^. The adsorption of carbon dioxide is a potential technique for removing CO_2_ from a gas mixture in commercial and industrial applications^4^. For anthropogenic CO_2_ collection, several methods have been developed, including amine–solvent scrubbing, cryogenic distillation, solid sorbents, membrane separation, and adsorption processes^5,6^. Because of its high overall efficiency, low power consumption, and low operating cost, as well as its ability to work across a wide range of temperatures and pressures, adsorption onto porous solid materials is a promising CO_2_ separation method^7^. Microfibers and microporous polymers have shown certain benefits over other adsorbents when used on various surfaces. Fibrous adsorbents, in particular, exhibit flexibility, minimal pressure drop, and a short transit distance^8^. Porous materials have been used as substrates for various amine-modified adsorbents since their high specific surface area, and pore volume is advantageous for increasing CO_2_ adsorption capacity^9^. Chemical and mechanical resistance are good in polymers. However, there are no chemical groups that are acceptable. As a result, functional groups must be bonded to their surfaces to turn them into excellent adsorbents. Using base polymer forms with a high surface ratio is one way to improve the bond percentage, which is why nanofibers are considered primary polymers^10^. Nanofibers have a specific surface area to volume ratio, surface tension, a low base weight, tiny scale pores, and a relatively high permeability^11^. Electrospinning is a generally reliable and straightforward method for producing nanofibers from a wide range of polymers^12^. As a result, many new research projects using electrospun nanofibers for various purposes have sprung up^13^. An efficient adsorbent of nanofiber/polyamide 6/carbon nanotube (PA/CNT) composite membrane composed of electrospun nanofibers coated with polyethylene (PEI) to adsorb carbon dioxide was produced by Zeinab and co-workers in 2017. Several basic characteristics, such as open porosity and excellent connection, are demonstrated. It has a 0.051 g/g adsorption capacity per gram of adsorbent. This adsorbent must be prepared at 105 degrees Celsius^14^. In the research of Olivieri and co-workers, the possibility of using an amine-activated nanofiber membrane for carbon dioxide adsorption was investigated. The electrospinning process was utilized to make nanofiber mattresses from poly (acrylonitrile) (PAN) powder. Hexa methylene di-amine or ethylene diamine were used for amination, followed by hydrolysis^15^. Many investigations have reported that chemically functionalizing fibrous or porous polymer substrates with an amine group for low temperature and humidity-aided adsorption. Dip-coating, interfacial polymerization, in situ polymerization, and graft copolymerization are some of the techniques for making polymeric membranes that have been developed. Radiation-induced graft (RIG) copolymerization is well-known for its advantages and potential for modifying pre-existing polymeric materials' chemical and physical characteristics without changing their intrinsic features^16–20^. The use of high-energy irradiation (gamma or electron beam) to modify polymers is well-known^21,22^. When polymeric materials are exposed to high-energy ionizing radiation, their chemical, physical (thermal, electrical, and optical), and mechanical characteristics are altered^23,24^. Lubna and co-workers used gamma radiation to bond vinyl acetate monomer (VAc) in various concentrations on recycled polyethylene terephthalate (r-PET) sheets^25^. Shamsi and co-workers developed a novel acid/base composite membrane, combining VP-4 bonding on PVDF films with PA doping under regulated circumstances. According to their findings, RIG is a straightforward and successful technique for producing membrane precursors with regulated physicochemical characteristics^26–28^. Yang and co-workers developed a solid amine adsorbent (PAN-AF) for CO_2_ adsorption by pre-bonding allylamine copolymerization on PAN fiber. This adsorbent has high thermal stability (about 220 °C), a high CO_2_ adsorption capacity (more than 6.22 mmol/g CO_2_), and a high PAN-AF regeneration temperature (around 100 °C). Allylamine poses a severe and hazardous risk to the environment^29^. In 2017, in a study by Rojek and co-workers, the microporous structure of ultrahigh molecular weight polyethylene (UHMWPE) followed by hydrolysis was presented to enhance the environmental aspects of adsorption and stability. As a result, the adsorbent with 108 grafting yields (GY) showed the highest adsorption capacity for CO_2_ at 0.0486 g/g. A monomer concentration of at least 80% is recommended for NVF^18^. Abbasi and co-workers created a nanofiber amine adsorbent for CO_2_ adsorption using electrospinning (s-PP), glycidyl methacrylate radiation, and ethanolamine amination in 2019. At 15 percent feed concentration at 30° C, the sample with a 300 percent bonding degree and 94 percent amination degree had a maximum adsorption capacity of 2.87 mmol/g. It demonstrates that the fiber adsorbent produced has a significant CO_2_ uptake capability. A temperature of 45 °C is required for s-PP electrospinning^19^.

This research developed radiation-grafting nano adsorbents that are capable of effectively adsorbing carbon dioxide at ambient temperature and pressure. Industrial polyacrylonitrile (PAN) electrospinning is prepared at ambient temperature and conditions with glycidyl methacrylate under simultaneous-irradiation method. The first type of amino groups of ethanolamine, the second type of diethylamine and the third type of triethylamine were used to functionalize ploy-GMA-PAN.

## Experimental

### Materials

The most critical materials used in the synthesis and production of polymeric adsorbents are shown in Table 1.

**Table 1:** Consumables in the synthesis of polymeric nanofibers under gamma irradiation.

Chemical name	Chemical formul	Molar Mas g/mol	Purity%	Chemical structure
Industrial Poly acrylonitrile	PAN	–	–	
N,N dimethylformamide (DMF)	C_3_H_7_ NO	73.09	99.99 ≥	
glycidyl methacrylate (GMA)	C_7_H_10_O_3_	142.15	99.00 ≥	
Ethanolamine (EA)	C_2_H_7_NO	61.08	99.00 ≥	
Diethylamine (DEA)	C_4_H_11_N	73.14	99.50 ≥	
Triethylamine (TEA)	C_2_H_15_N	101.19	99.00 ≥	
Methanol	CH_3_OH	32.04	99.00 ≥	
Carbon dioxide gas capsule	CO_2_	44.0095	99.99 ≥	

### Polymeric adsorption bed synthesis

**Figure 1:**
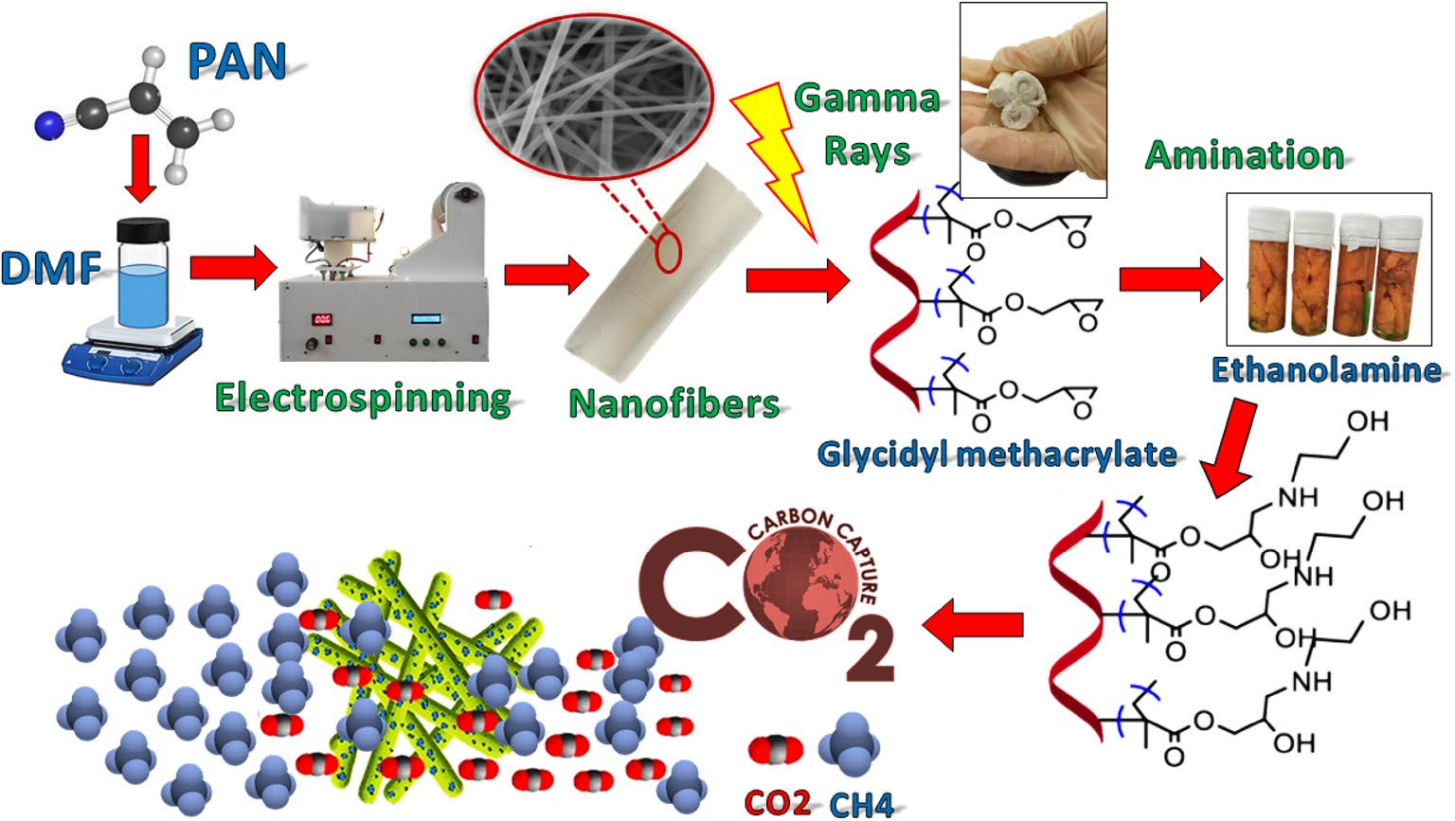
Schematic procedure for the preparation of the polymeric adsorbents.

The schematic procedure for the preparation of the polymeric adsorbents is shown in Fig. 1. The adsorption synthesis consists of the electrospinning of polyacrylonitrile, the radiation grafting of nanofibers, and the modification procedure, and the experimental tests with CO_2_/CH_4_ gases.

#### Electrospinning PAN

The mixture of industrial PAN and DMF solvent was placed on a magnetic stirrer for 12 h to obtain a uniform solution. Pour the PAN solution into a 5 ml syringe and place it on the syringe pump. Laboratory-scale automatic electrospinning machine and single-syringe rotation system were electrospinning at a relative humidity under ambient conditions. The nanofiber membrane is collected from a metal roller and placed in an oven at 45 °C for 3 h to allow the solvent to evaporate well. Table 2 shows the electrospinning nanofibers under different operating conditions.

**Table 2:** PAN electrospinning in different operating conditions.

Run	Distance (cm)	Flow rate (ml/h)	Voltage (Kv)	(wt%)
1	10	1	10	5
2	15	1	10	5
3	20	1	10	5
4	10	1	10	10
5	15	1	10	10
6	20	1	10	10
7	10	1	10	15
8	15	1	10	15
9	20	1	10	15
10	15	2	10	15
11	15	1	15	15
12	15	2	15	15
13	15	1.5	15	15

#### GMA bonding on PAN nanofibers simultaneous irradiation

PAN substrate synthesis was prepared by the simultaneous-irradiation method by gamma-ray. The synthesized PAN nanofibers were irradiated in different doses (10–50 kGy). The concentration of GMA monomer is 5–20% diluted with methanol. The adsorbents are placed inside the glass and sealed well. Nitrogen gas (N_2_) is injected for 10 min for cleansing and purification. The samples are immersed in the solution overnight and then irradiated. To eliminate non-reactive monomers and potential homopolymers, the bonded samples were washed multiple times with methanol. Then put it in the oven at 50 °C for 12 h to dry completely. The grafted samples are weighed to calculate the degree of grafting (% DG), as shown in Eq. (1)^30^.

1 $$\left( {DG\% } \right) = \frac{{W_{f} - W_{i} }}{{W_{i} }} \times 100$$

W_f_ and W_i_ are the initial and final weights after grafting, respectively.

#### Amination PAN irradiated with GMA

Poly-GMA-PAN participated in various functional reactions with the three amines EA, DEA and TEA. The amination reaction was performed by immersing the GMA-bound PAN nanofibers in EA (60/40%) (v/v) with water to obtain a uniform solution, in a shaker at 60 °C for 4 h. For DEA and TEA, the ratio was (60/40%) (v/v) with water, which was placed in a shaker at different times of 4 and 24 h at different temperatures of 60 °C and 30 °C, respectively. The samples were then washed thoroughly with deionized water to remove non-reactive amines. Modified samples were placed in an oven at 50 °C for 4 h. The modified samples were weighted to calculate the amination percentage (% DA) with Eq. (2)^31^.

2 $$\left( {DA\% } \right) = \frac{{\frac{{W_{a} - W_{f} }}{{MW_{a} }}}}{{\frac{{W_{f} - W_{i} }}{142.15}}} \times 100$$

In Eq. (2), W_i_, W_f_, W_a_, MW_a_ are the prototype weight, sample weight after grafting, sample weight after amination, and the molecular weight of amine as well as GMA molecular weight ~ 142.15, respectively.

#### Characterization methods

The morphology of the final adsorbent and the different stages of its preparation was examined using a scanning electron microscope (SEM) model (Hitachi Su3500) after gold coating. Transplantation and amination reactions of polymeric adsorbents were confirmed by FTIR (Bruker, victor22) spectroscopy.

#### *CO*_*2*_* adsorption test*

A CO_2_ analyzer determined the CO_2_ adsorption test with a nondispersive infrared sensor (NDIR) range (0–30%). The CO_2_ and CH_4_ gas flows are controlled by two MFC mass flow controllers in the range (1–100%) with a maximum accuracy of 1% of the final range of the device plus 1% of the regulated current. The adsorption column is made of Plexiglas with a height of 200 mm and a diameter of 10 mm. The adsorption test was performed with 1 g of synthesized adsorbent punched and placed in the column. Fiberglass was used to hold the adsorbent at the beginning and end of the column. To control the temperature around the adsorption column, a heating coil was wound. A mixture of CO_2_ and CH_4_ gas with a total flow rate of 100 ml/min with a concentration range of CO_2_ (5%) at atmospheric pressure was used for adsorption experiments at ambient temperature. The adsorption capacity of the samples is estimated using Eq. (3)^32^.

3 $$Q = \frac{{F \times C_{0} \times t_{q} }}{W}$$

In the above equation, F, C_0_, and W are the total flow rate (mmol/s), initial concentration, and adsorbent weight (g). The stoichiometric time (t_q_) versus second is also calculated by Eq. (4)^32^.

4 $$t_{q} = \int_{0}^{t} {\left( {1 - \frac{c}{{c_{0} }}} \right)} dt$$

**Figure 2:**
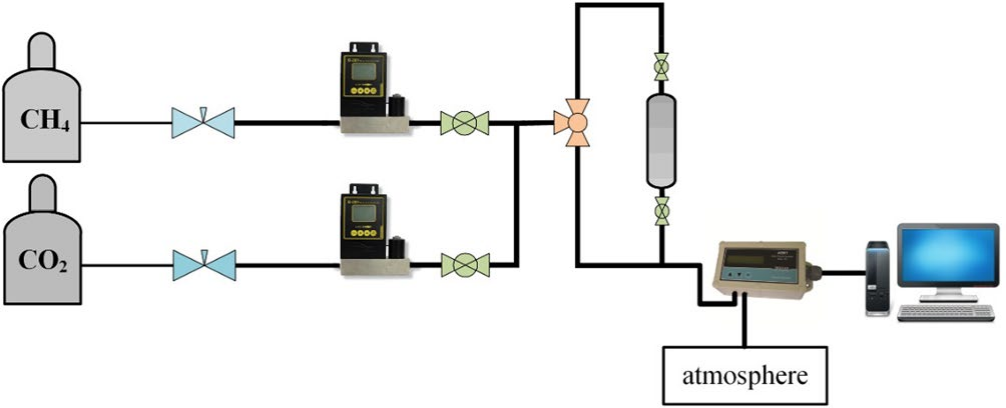
Schematic diagram of fixed-bed adsorption system.

In Eq. (4), C_0_, C is the concentration of CO_2_ in the gas inlet and outlet, respectively. The schematic diagram of the fixed-bed adsorption system is shown in Fig. 2.

## Results and discussion

### Morphology and analysis of SEM

**Figure 3:**
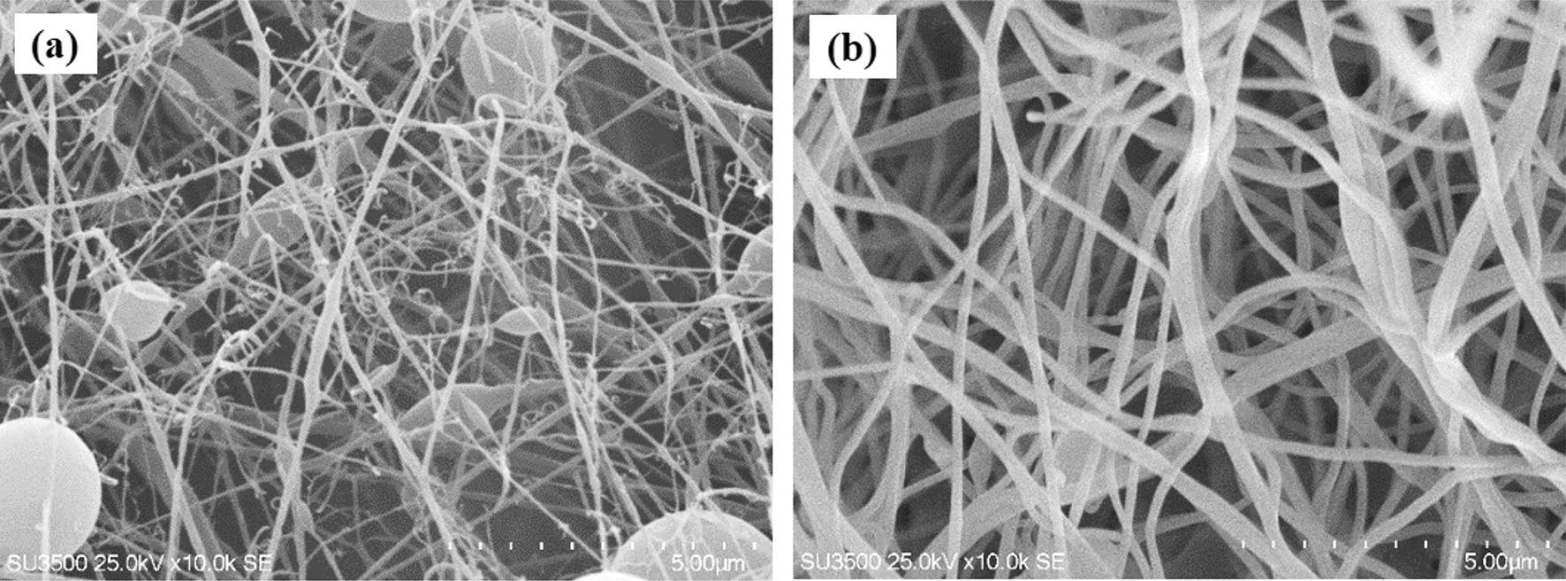
SEM images at concentrations (5–10% wt), distance (15) and voltages (15 kV).

PAN electrospinning was performed by three parameters of voltage, weight percentage and needle to collector distance. At a concentration of 5% wt at intervals of 10, 15 and 20 cm needle to collector distance, low viscosity and accumulation of solvent caused the formation of many droplets, as well as irregular morphology. Figure 3a shows the drops and irregularities at a distance of 15 cm. At 10% wt concentration, better fibers are obtained, at 10 cm distance, the fibers have less droplets, also at 15 cm distance, the fiber density is more suitable, which decreases with increasing distance and the effect of low voltage causes more knots (as shown in Fig. 3b). By increasing the viscosity at 15% wt at a distance of 10 cm, the high concentration of the solution and the low evaporation time of the DMF solvent caused the formation of drops and tears. The increase in distance has caused irregularities in fiber formation. At a feed rate of 2 ml/h, increasing the concentration of the solution at a distance of 10 cm and applying low voltage causes smaller diameters. At a concentration of 15% wt, fibers with a suitable diameter are obtained, which at a voltage of 15 kV, the lower diameter of the fibers indicates the ideal electrospinning conditions. Optimal feed rate conditions of 1.5 ml/min have saved time and created fibers with suitable diameter, morphology, and thickness, so the synthesis of the adsorbent bed has been done with these conditions.

**Figure 4:**
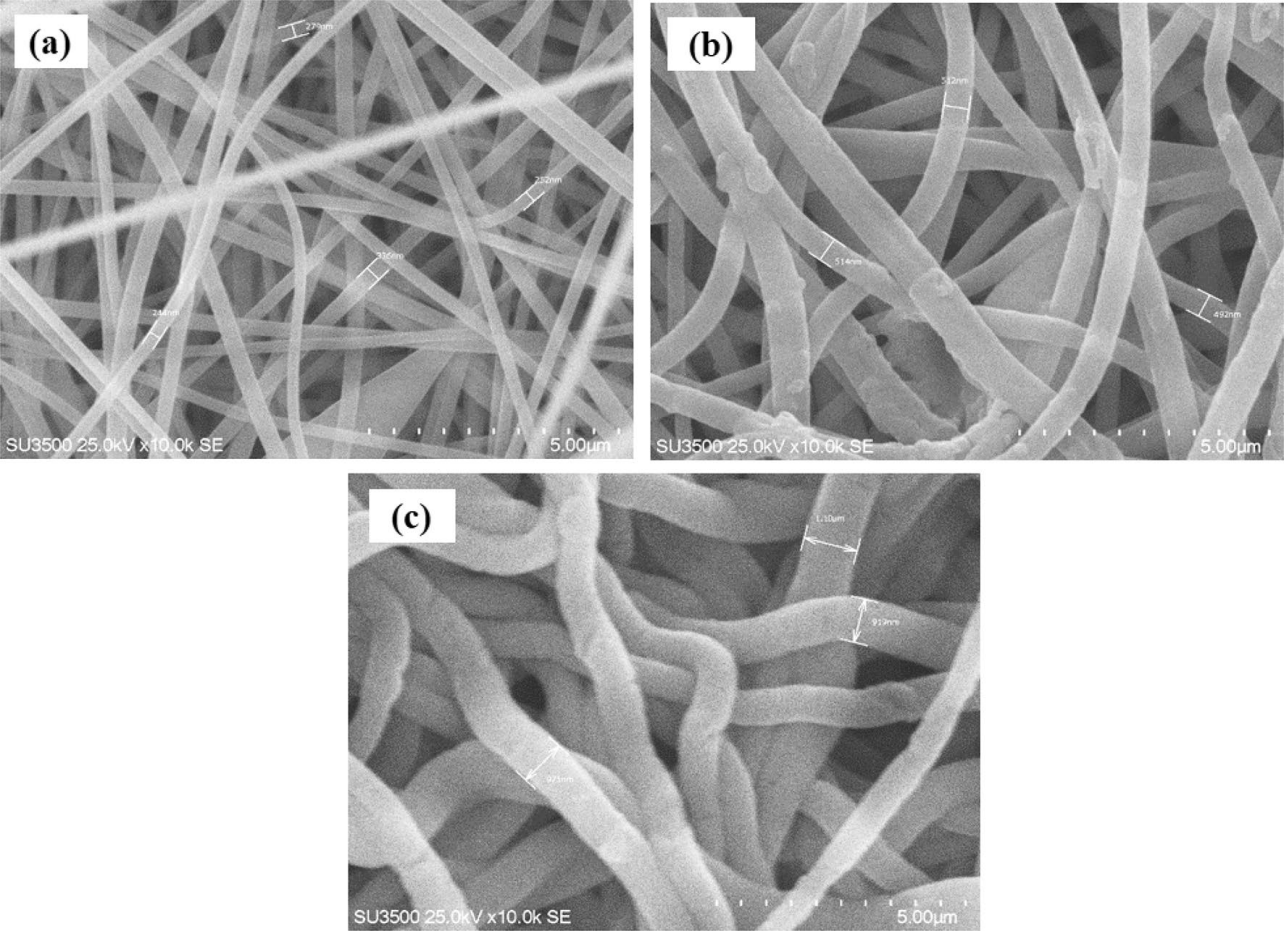
SEM images of (**a**) PAN nanofibers; (**b**) GMA grafted nanofibers; (**c**) EA aminated nanofibers PAN.

The SEM images in Fig. 4 showed the average diameter of the fibers. The diameters were randomly selected with the original software. In Fig. 4a, PAN crude nanofibers are shown with an average diameter between 245 and 350 nm. After the bonding reaction during the PAN-NF irradiation process with GMA, the average diameter of the fibers has reached 450–515 nm (as shown in Fig. 4b), which increases in diameter. The grafting proves the success of the GMA transplant, so the maximum grafting percent is obtained equal to 211.14 DG%. In Fig. 4c, the mean diameter of poly (GMA) Grafted PAN increased after amination with ethanolamine to 919 nm–1 μm, indicating the proper performance of the amination reaction and the formation of amino group layers on PAN-g-GMA-EA nanofibers.

### Chemical properties of the adsorbent

**Figure 5:**
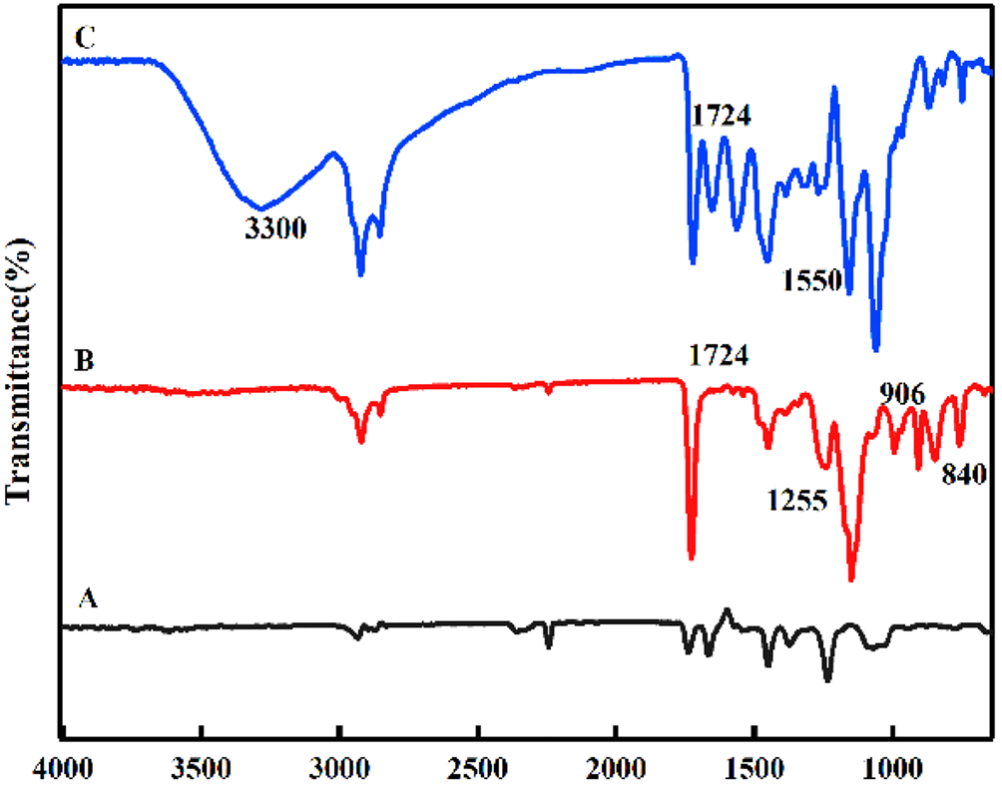
FTIR Spectra of (**A**) PAN nanofibers; (**B**) GMA grafted nanofibers and (**C**) EA aminated nanofibers with 60%

The FT-IR peaks of the polymeric adsorbents are shown in Fig. 5. The GMA bond reaction showed the main peaks at 840 cm^−1^, 906 cm^−1^, 1255 cm^−1^ and 1724 cm^−1^. The two small peaks 840 and 906 are related to the epoxy and co groups, respectively.

Strong tape at 1724 cm^−1^ and weak wide tape at 1255 cm^−1^ are acrylate tensile profiles –C=O and C–O–, respectively. As can be seen in Fig. 5, after the amination reaction with the ethanolamine peaks of the epoxy groups almost disappeared, the intensity of the P-GMA carbonyl bond in 1724 cm^−1^ did not change significantly during the amination reaction. In addition, the N–H tensile band appeared in the range of 3300–3500 cm^−1^ and in the range of 1350–1000 cm^−1^ C–N band as well as the C–C band in the range of 1550–650 cm^−1^ after amination. The adsorption of carbon dioxide falls into two main categories: chemical adsorption and physical adsorption, given that in many cases the mechanism of chemical adsorption keeps carbon dioxide molecules stronger than the mechanism of physical adsorption. So, they know chemical adsorption is more effective. In addition, the adsorption of carbon dioxide in porous materials depends on the surface active sites and the porosity of the substrate used. Because our adsorbent is porous, it is functionalized during the amination reaction after grafted GMA with amine groups. The amine groups in the synthesized adsorbent react with the carbon dioxide molecules to form carbon dioxide amine complexes. The possible reaction between the amine group and the carbon dioxide molecules is shown in the following Eq. (1).

5 $${\text{CO}}_{2} + 2{\text{RNH}}_{2} \leftrightarrows {\text{RNHCOO}}^{ - } + {\text{RNH}}_{3}^{ + }$$

6 $${\text{CO}}_{2} + 2{\text{R}}_{2} {\text{NH}} \leftrightarrows {\text{R}}_{2} {\text{NH}}_{2}^{ + } + {\text{R}}_{2} {\text{NCOO}}^{ - }$$

7 $${\text{CO}}_{2} + 2{\text{R}}_{3} {\text{N}} \leftrightarrows {\text{R}}_{4} {\text{N}}^{ + } + {\text{R}}_{2} {\text{NCOO}}^{ - }$$

### Adsorption performance

The performance of the PAN adsorbent was evaluated using CO_2_, CH_4_ in a mixture containing 5% CO_2_ at atmospheric pressure and ambient temperature.

#### Effect GMA concentration on (DG%) and adsorption capacity

**Figure 6:**
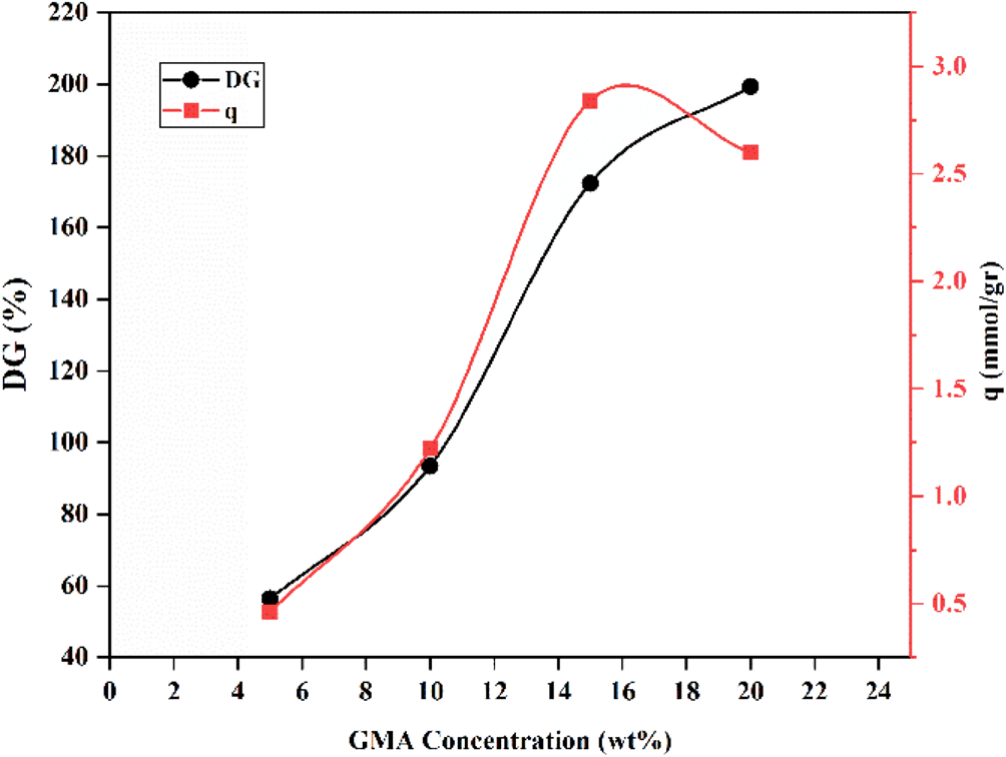
Effect of GMA concentration on (DG%) and adsorption capacity (q).

Figure 6 shows the effect of the GMA monomer concentration parameter on the adsorption capacity and the degree of grafting (DG%). In addition, the radiation dose and amine concentration are set to 20 kGy and 65%, respectively. As the concentration of GMA increased from 5, 10, 15 and 20 wt%, the degree of grafting (DG%) increased from 56.33 to 199.32. The polymerization reaction increased with the increase of GMA concentration from 5 to 15 wt%, because free radicals had easier access to monomer molecules. Due to the creation of possible homopolymers and the interruption of reactive chains, increasing the concentration of GMA from 15 to 20% by weight leads to the cross-linking effect on the carbon dioxide adsorption capacity, and the adsorption capacity has decreased from 2.84 to 2.61 mmol/g.

#### Effect of radiation dose on (DG%) and adsorption capacity

**Figure 7:**
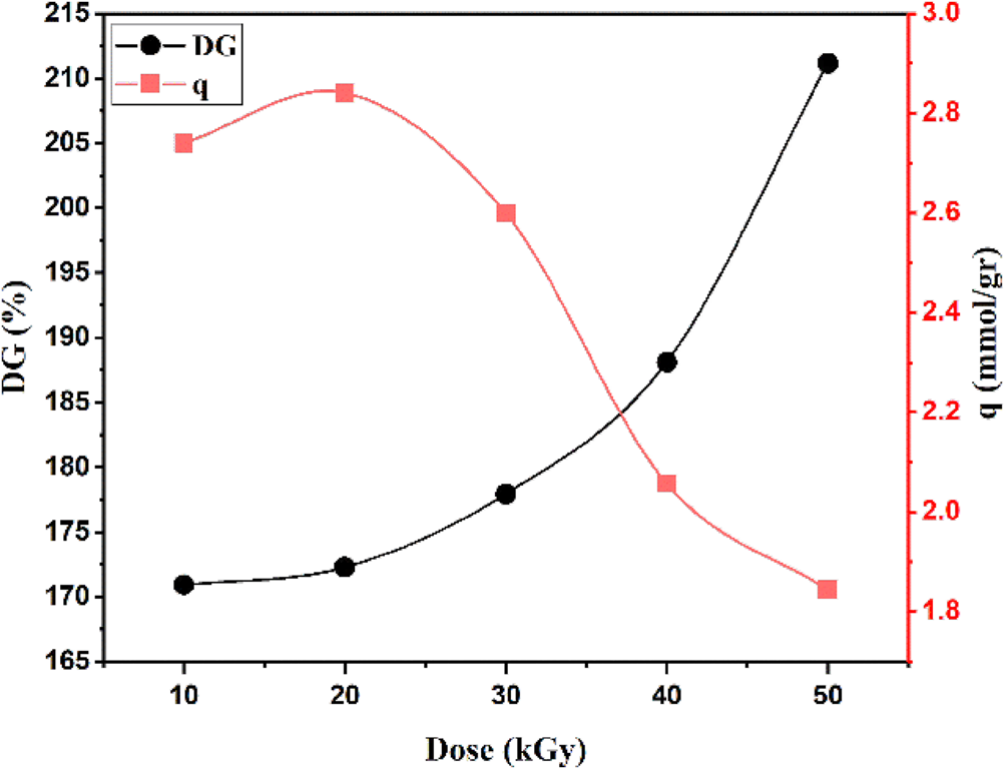
Effect of radiation dose on (DG%) and adsorption capacity (q).

As shown in Fig. 7, with increasing radiation dose from 10 to 50 kGy, the degree of bonding increased by 40.23%. As the radiation dose is increased from 10 to 20 kGy DG% and DA%, the adsorption capacity is also increased, which can be attributed to the release of more radicals and the creation of more active sites for grafting, which results in a higher adsorption capacity. At higher irradiation doses, homopolymer formation reduces the active sites for amination.

As you can see in Fig. 7, the highest adsorption capacity is 2.84 mmol/g at a dose of 20 kGy and the degree of grafting is 172.26% DG. This indicates that increasing the radiation dose increases the degree of grafting. The adsorption capacity increases from 10 to 30 kGy with increasing radiation dose and then decreases from 30 to 50 kGy.

Table 3, shows a comparison of other studies in the field of CO_2_ uptake. This study introduces PAN adsorbent synthesized by simultaneous-irradiation method and then amination to adsorb CO2. An adsorbent's advantages include its economical productivity due to its use of industrial polymers, its ease of synthesis, its effective adsorption, and its ease of regeneration.

**Table 3:** Polymeric adsorbents for the removal of CO_2_ gas.

Polymer	Monomer	Irradiation method	Functional Agent	Type adsorbent	Time/Temperature regeneration	CO_2_%	Adsorption Capacity	REF
PE	Acrylice Acid	Pre-irradiation UV-grafting	PEI	Membrane	–/80	–	–	^40^
UHMWPE	N-VinylFormamid	Pre-irradiation Electron bean	Hydrolysis with NAOH	Film	15/80	10	0.0486 g/g	^41^
PP	GMA	Pre-irradiation. Electron bean	EA DEA TEA	Fiber	15/80	5/10/15	2.87 mmol/g	^19^
PE-PP	GMA	Pre-irradiation. Electron bean	TEA	Nonwoven fiber	40/80	–	4.52 mmol / g	^18^
PAN	Allylamine	Pre-irradiation Gama ray	(NH_4_)_2_S_2_O_8_/NaHSO_3_	Fiber	30/100	5–15%	6.22 mmol/g	^29^

#### Effect of temperature, amine concentration and radiation dose on the degree of amination

**Figure 8:**
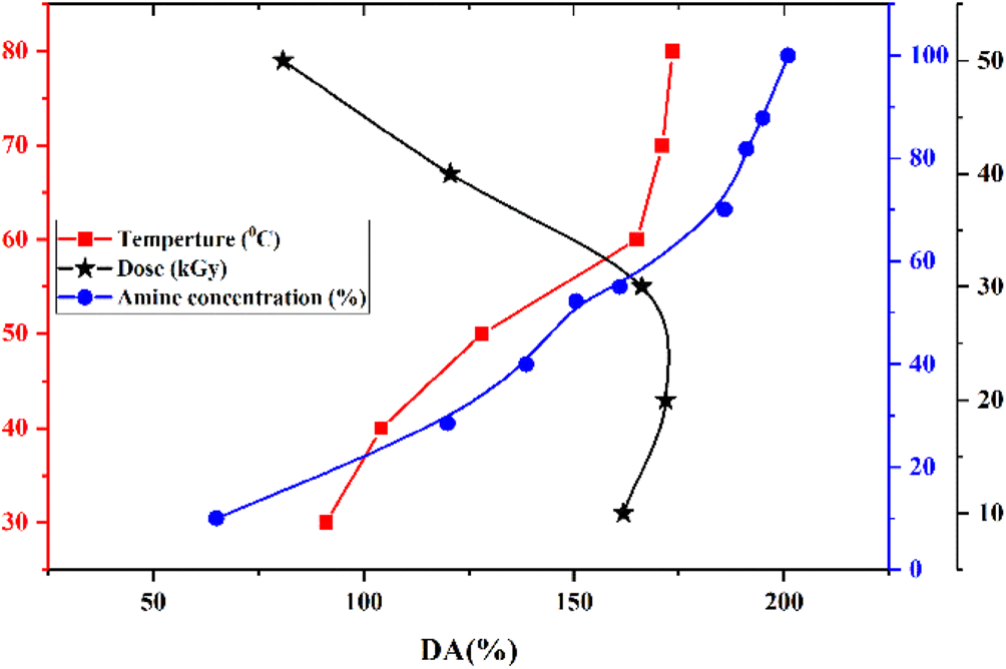
Effect of temperature, amine concentration and radiation dose on the degree of amination (DA%).

Figure 8 shows the amination reaction at 30–100 °C and a constant concentration of 65% vol in water, and a constant radiation dose of 20 kGy. With increasing temperature from 30 to 60 °C, DA ٪ increased with a steep slope from 91.15% DA to 165 ٪ DA. This trend of change was lower with increasing temperature from 60 to 80 °C, by 7.87%. Also at a temperature of 60° C and a constant radiation dose of 20 kGy, when the amine concentration changed from 10 to 65%, the degree of amination increased from 65% DA to 170.83% DA. Due to the reaction of more amine molecules in contact with epoxy groups attributed. From 65 to 100% by volume of amines the slope of this process has decreased significantly and reached 15.07%. Which can be attributed to the filling of active amine sites. Figure 8 also shows the relationship between radiation dose and DA% from 10 to 50 kGy at constant concentrations and temperatures of 65% and 60° C, respectively. At 10–20 kGy, increasing the degree of bonding increased the DA%, then dropped from 30 to 50 kGy and finally reached 80% DA.

#### Assess adsorption capacity

**Figure 9:**
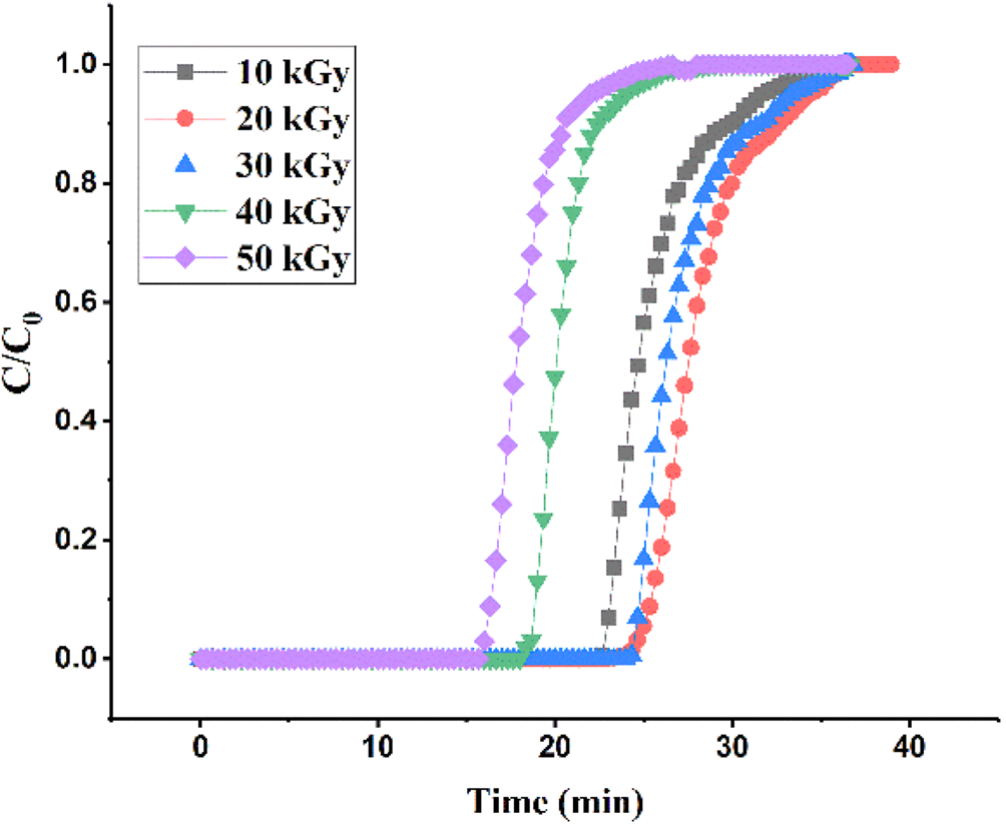
Breakthrough curves of CO_2_ adsorption.

In Fig. 9, as shown, the PAN -g -GMA -EA adsorbent shows an increase in adsorption capacity by increasing the irradiation dose of the Breakthrough curve. This trend can be attributed to the increase of DA%. In fact, more amine sites are available to capture CO_2_ at 20 kGy. From 30 to 10 kGy, respectively, the decreasing trend of DA% has slightly reduced the adsorption capacity. Also, a reduction slope in the adsorption capacity from 40 to 50 kGy has been created due to the reduction of DA%.

Adsorbents modified with DEA and TEA had low adsorption capacities of 1.58 and 0.63, due to low amination degrees, 149.16% and 107.87%, respectively.

#### Breakthrough curve modelling

The parameter design of the fixed-bed column and its practical industrial scale application are implemented using mathematical modelling of BTCs. Adsorption models often were used to predict the adsorptive curve as well as express relative adsorptive behavior. As a consequence, five simple models (Table 4) are used to evaluate the dynamic characteristics of PAN-g-GMA-EA adsorbents in the column, estimate relative determinants, and examine the outcomes of column adsorption tests.

**Table 4:** Various mathematical models for breakthrough curve prediction.

Column adsorption model	Equation	Plot	Parameter of model
Thomas model (TM)	$\frac{c}{{c_{0} }} = \frac{1}{{1 + e^{{\frac{{k_{Th} q_{0} m}}{Q} - k_{Th} t}} }}$	Versus time $\frac{c}{{c_{0} }}$	$k_{Th}$ $q_{0}$
Yoon–Nelson model (YNM)	$\frac{c}{{c_{0} }} = \frac{{e^{{k_{YN} t - \tau k_{YN} }} }}{{1 + e^{{k_{YN} t - \tau k_{YN} }} }}$	Versus time $\frac{c}{{c_{0} }}$	$k_{TY}$ T
Adam and Bohart model (ABM)	$\frac{c}{{c_{0} }} = \frac{1}{{e^{{K_{AB} N_{0} \frac{z}{u} - K_{AB} C_{0} t}} + 1}}$	Versus time $\frac{c}{{c_{0} }}$	$k_{AB}$ $N_{O}$
Clark model (CM)	$\frac{c}{{c_{0} }} = \left( {\frac{1}{{1 + Ae^{ - rt} }}} \right)^{1/(n - 1)}$	Versus time $\frac{c}{{c_{0} }}$	$A$ $R$
Yan model (YM)	$\frac{c}{{c_{0} }} = 1 - \frac{1}{{1 + \left( {Qt/b} \right)^{a} }}$	Versus time $\frac{c}{{c_{0} }}$	$a$ $b$

#### Thomas model

**Figure 10:**
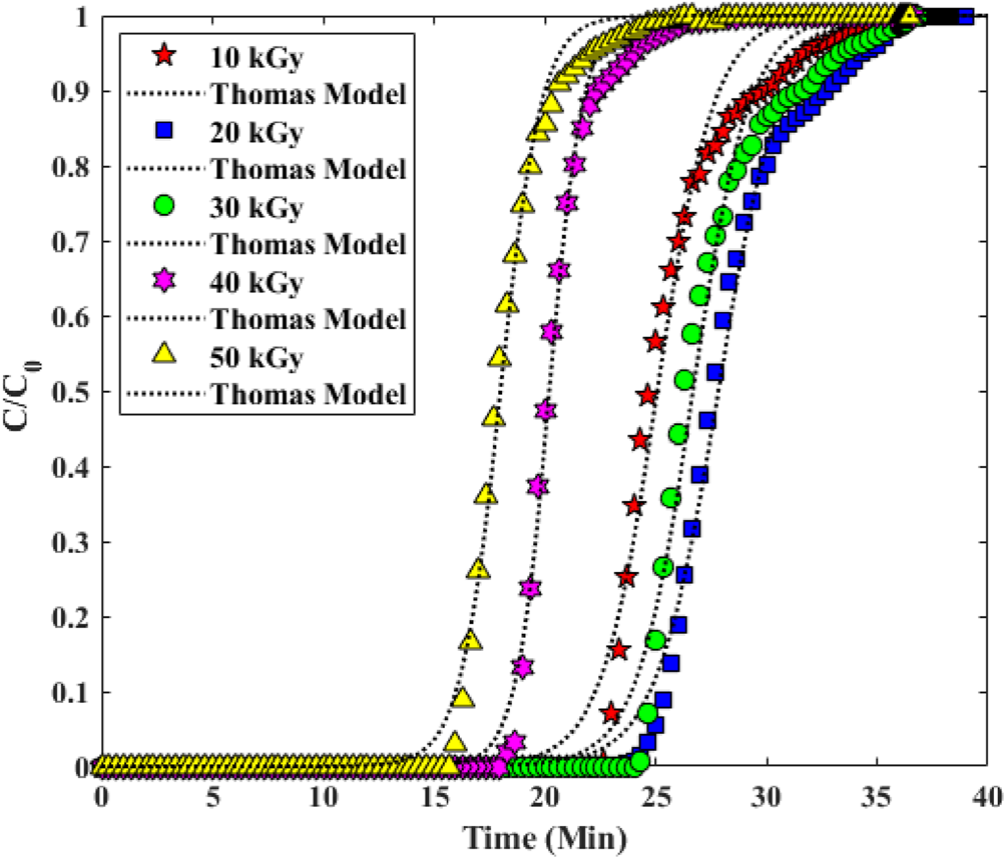
Investigation of Experimental Breakthrough curves in columns with Thomas model.

Evaluation of the progress curves and adsorption capacity of FBACs by Thomas model is particularly useful for predicting the amount of adsorbent saturation due to the relationship between (C/C_0_) and the input time, especially for adsorption. Based on the results of nonlinear fitting, the correlation is high (R^2^ > 0.99). Experimental data and model indicated that the process of CO_2_ uptake on PAN-g-GMA-EA can be well interpreted. Breakthrough curve with Thomas model, as shown in Fig. 10. The constants and parameters of the Thomas model are shown in Table 5^33,34^.

**Table 5:** Fitting results and main parameters of the models of Thomas, Clark,Yoon-Nelson, Bohart Adam and Yan.

C_0_	Flow rate	Dose (kGy)	qexp	qm	kTh	R_2_	SSE	RMSE
Thomas model								
5	100	10	120.59	121.1	16.32	0.9948	0.1487	0.0336
5	100	20	124.99	121.4	16.31	0.998	0.0892	0.0213
5	100	30	114.38	114.7	18.39	0.9942	0.1568	0.0347
5	100	40	90.53	96.29	27.98	0.9986	0.043	0.0185
5	100	50	8.15	83.49	23.64	0.9984	0.4417	0.0191

C_0_	Flow rate	Dose (kGy)	R^2^	SSE	RMSE			
Clark model								
5	100	10	0.998	0.0588	0.0211			
5	100	20	0.9994	0.0265	0.0116			
5	100	30	0.9976	0.0644	0.0225			
5	100	40	0.9996	0.0109	0.0093			
5	100	50	0.9997	0.0074	0.0078			

C_0_	Flow rate	Dose (kGy)	kYn	τ	R^2^	SSE	RMSE	
Yoon-Nalson								
5	100	10	0.7871	25.08	0.9948	0.1487	0.0334	
5	100	20	0.7112	27.83	0.998	0.0892	0.0213	
5	100	30	0.7905	26.7	0.9942	0.1568	0.0346	
5	100	40	1.333	20.21	0.9986	0.043	0.0184	
5	100	50	1.096	18.01	0.9984	0.0442	0.019	

C_0_	Flow rate	Dose (kGy)	N_0_	k_AB_	R2	SSE	RMSE	
A-B model								
5	100	10	36.59	22.57	0.9948	0.1487	0.0338	
5	100	20	21.03	11.67	0.998	0.0892	0.0214	
5	100	30	31.81	12.34	0.9942	0.1568	0.035	
5	100	40	20.09	23.21	0.9986	0.043	0.0186	
5	100	50	29.65	11.37	0.9984	0.0442	0.0193	

C_0_	Flow rate	Dose (kGy)	A	B	R^2^	SSE	RMSE	
Yan model								
5	100	10	19.27	3075	0.9958	0.1203	0.0302	
5	100	20	19.55	3426	0.9985	0.0667	0.0184	
5	100	30	20.61	3043	0.9952	0.1295	0.0316	
5	100	40	26.56	1869	0.9988	0.0345	0.0166	
5	100	50	19.43	1423	0.9989	0.0312	0.0161	

#### Bohart and Adams model

**Figure 11:**
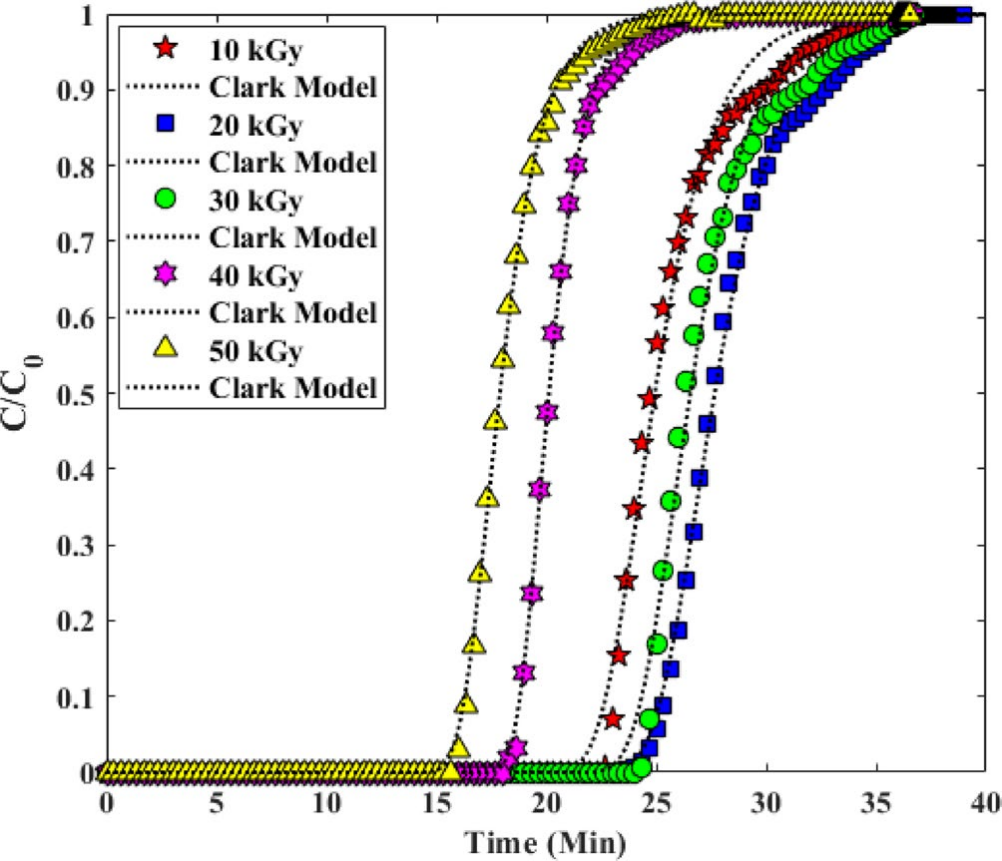
Investigation of Experimental Breakthrough curves in columns with Clark model.

In 1920, the first fixed bed dynamics analysis was performed by Bohart and Adams, who evaluated the uptake of chlorine gas in fixed bed columns filled with charcoal. Their model assumes that the adsorbent is irreversibly adsorbed as well as the local removal rate, which is proportional to the residual capacity of the adsorbent and the gas phase concentration of the adsorbent. Interestingly, Bohart and Adams predicted the correct form of progress analytically, but did not use any empirical data to test their equation. The Adam’s and Bohart(A-B) model is shown in Table 4. The constants and parameters of the A-B model are obtained in Table 5. The fit of the Breakthrough curve with model A-B is shown in Fig. 11^35,36^.

#### Clark model

**Figure 12:**
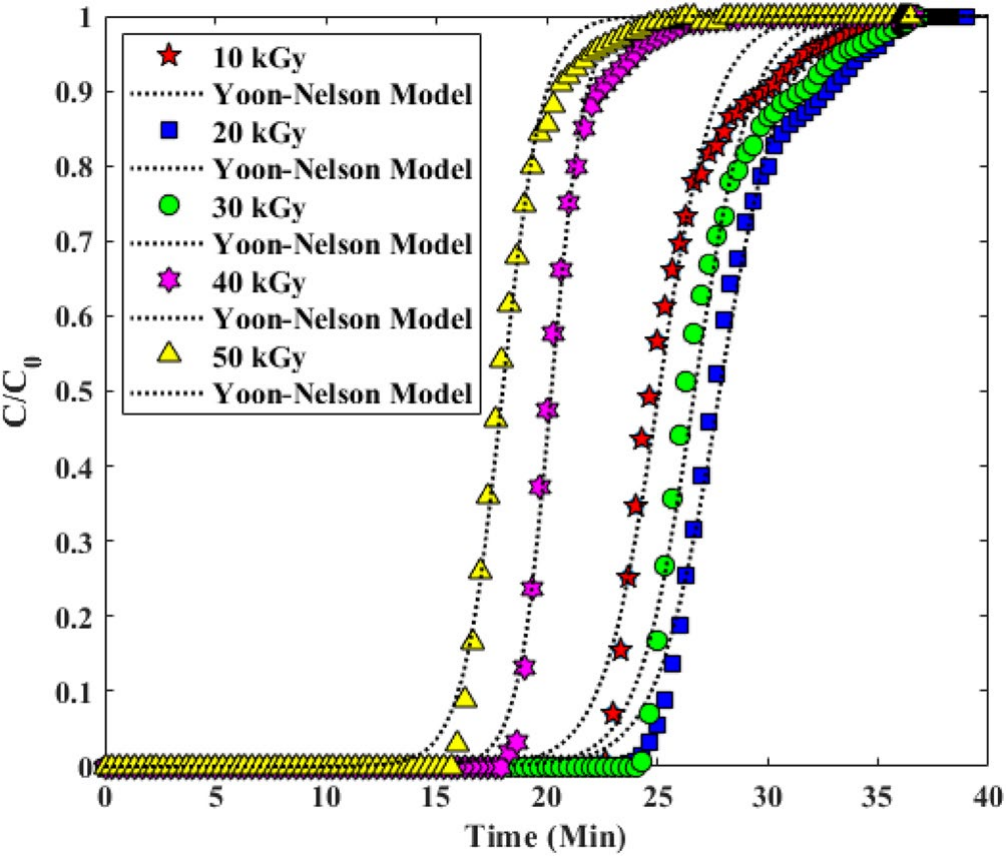
Investigation of Experimental Breakthrough curves in columns with Yan-Nelson model.

In the literature, which has been studied and reported for FBAC, the Clark model is of particular importance among the various models. Because Clark's model considers the nature of the mass transfer curves and equilibrium adsorption curves to predict the results (as shown in Table 4). The result of the linear curve fitting of the progress curves by the Clark model is shown in Fig. 12. The assumptions of Clark model are: (1) In a column, the nature of the flow is piston type. (2) Freundlich isotherm. Table 5 shows the constants and parameters (CM)^34,37^.

#### Yoon–Nelson model

**Figure 13:**
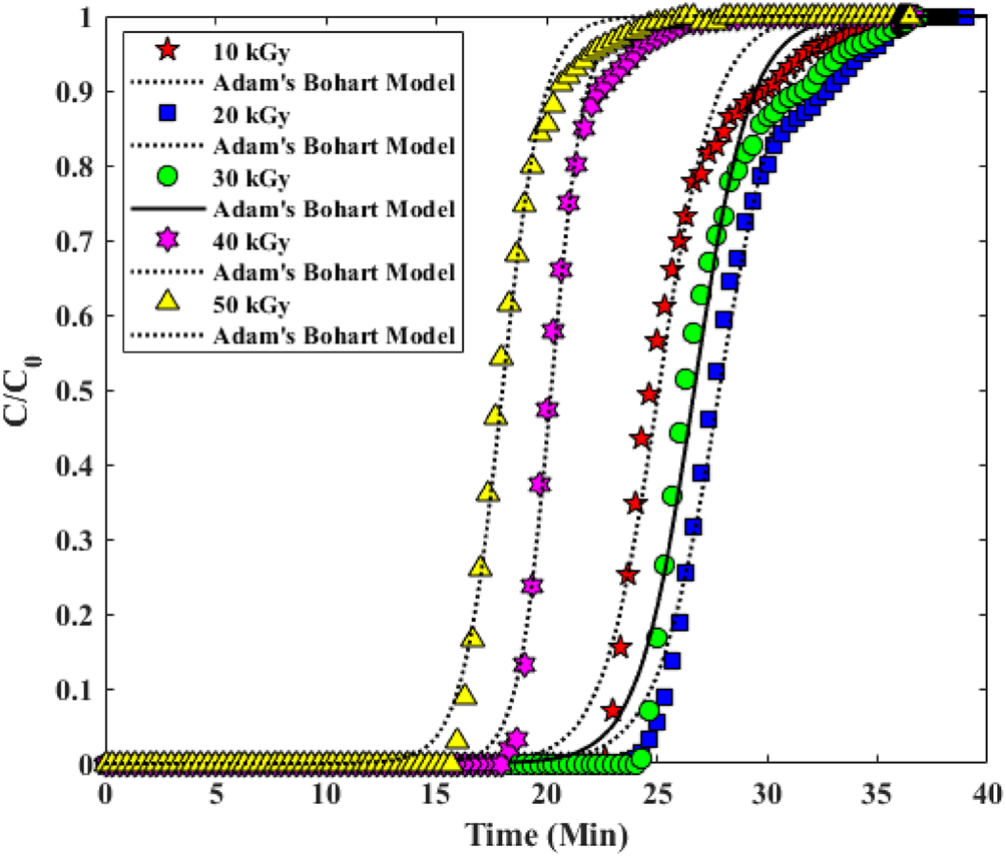
Investigation of Experimental Breakthrough curves in columns with Adams-Bohart model.

One of the simple models that has been proposed to study the adsorption of a continuous column with a fixed bed is the Nelson ion model (as shown in Table 4). The Y-N model assumes that for each adsorbent molecule the rate of reduction of the adsorption probability is proportional to the probability of adsorption of the adsorbent and the probability of the adsorbent advancing on the adsorbent. The various evaluated parameters of the Y-N model are reported in Table 5. Also with respect to R^2^ > 0.99 it can be reported that the experimental data of the column study fit well with the model, as shown in Fig. 13^33,38^.

#### Yan model

**Figure 14:**
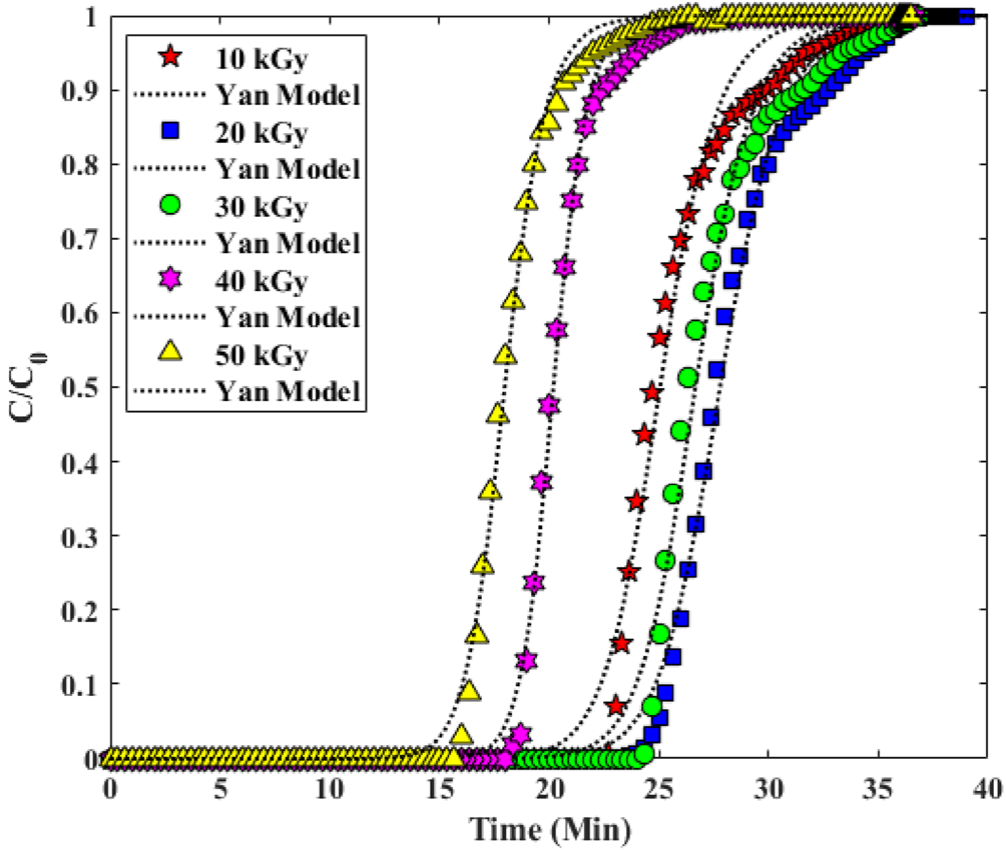
Investigation of Experimental Breakthrough curves in columns in with Yan model.

Another common model for examining column adsorption Breakthrough curves is the Yan model. At the beginning and end of the column adsorption Breakthrough curves by the Thomas model, there was an inaccuracy, so the Yan model was used to compensate for the overestimated values of the Thomas model adsorption capacity. According to the results of fitting the curve of Fig. 14, it can be claimed that according to the experimental data and the correlation coefficient R^2^, this model has done a good evaluation, which is specified in Table 4 of the constants a, b and the obtained results in Table 5^34,37,39^.

#### Stability of nanofibers PAN adsorbent

**Figure 15:**
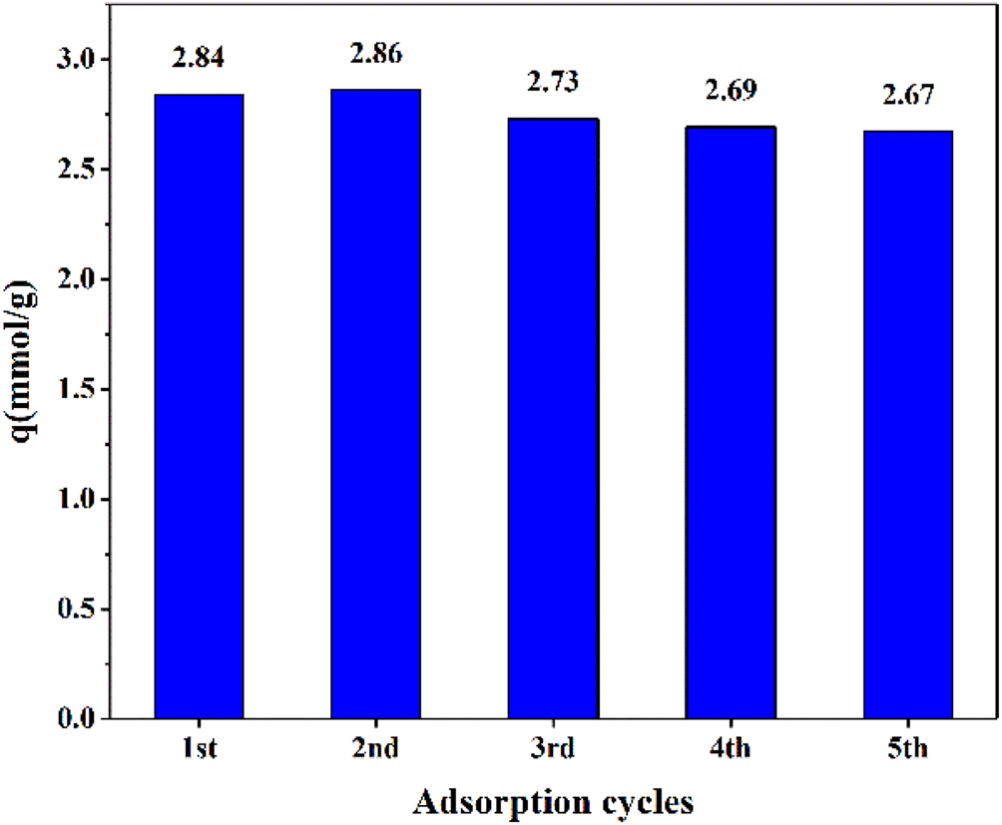
CO_2_ PAN adsorption capacity in a number of adsorption–desorption cycles.

Figure 15 shows the changes in the adsorption capacity of PAN adsorbent carbon dioxide. Excellent stability of the amine-containing adsorbent has been shown after four adsorption–desorption cycles despite similar breakthrough. As the adsorption capacity increased in the first cycle, it reached 2.84 mmol/g to 2.86 mmol/g. This increase in adsorption capacity can be attributed to the loss of moisture in the adsorbent with increasing temperature. After the second cycle, a slight decrease was observed and the adsorption capacity decreased to 2.73 mmol/g. This decrease was smaller in subsequent cycles, reaching 2.69 mmol/g in cycle 4. Therefore, it can be concluded that no significant change occurred in the mass transfer region.

## Conclusion

Due to the increasing industrial growth and global climate change, the need to isolate and adsorb greenhouse gases has received particular attention. Among these, adsorbents that are prepared with low consumption and low energy are one of the basic needs. The science of nanotechnology is one of the things that has opened the eyes in this field. This study used electrospinning ambient conditions to prepare a PAN adsorbent substrate for CO_2_ adsorption. Adsorbents were prepared in subsequent steps including PAN, RIG with GMA and amination with DEA, EA and TEA. Also, using FTIR and SEM tests, the bonds obtained from the amination reaction, the morphology of the synthesized adsorbent and the strength of the synthesized adsorbent were evaluated, respectively. The amination reaction was performed by immersing the GMA-bound PAN nanofibers in EA (60/40%) (v/v) with water, in a shaker at 60 °C for 4 h. The adsorption capacity was 2.84 mmol/g for the sample was 172.26% DG and 170.83% DA, which was obtained at a flow rate of 100 ml/min with a CO2 ratio of 5% to 95% CH4. Mathematical models including Thomas, Bohart and Adams, Clark, Yoon-Nelson, and Yan were used to analyze the experimental results. Also, this adsorbent is synthesized from 5 adsorption–desorption cycles, which shows good stability at a temperature of 80 °C and a short time of 15 min, and the need for low energy of this adsorbent is one of its special advantages.

## Data Availability

The datasets used and/or analysed during the current study available from the corresponding author on reseanable request.

## References

[1] Zainab G, Babar AA, Iqbal N, Wang X, Yu J, Ding B (2018). Amine-impregnated porous nanofiber membranes for CO2 capture. Compos. Commun..

[2] Ge K, Yu Q, Chen S, Shi X, Wang J (2019). Modeling CO2 adsorption dynamics within solid amine sorbent based on the fundamental diffusion-reaction processes. Chem. Eng. J..

[3] Abouelella DM, Fateen S-EK, Fouad MM (2018). Multiscale modeling study of the adsorption of co2 using different capture materials. Evergreen Joint J. Novel Carbon Resour. Sci. Green Asia Strategy.

[4] Saiwan C, Muchan P, deMontigny D, Tontiwachwutikul P (2014). New poly (vinylbenzylchloride/divinylbenzene) adsorbent for carbon dioxide adsorption: II: Effect of amine functionalization. Energy Procedia.

[5] An S, Lee JS, Joshi BN, Jo HS, Titov K, Chang JS, Jun CH, Al-Deyab SS, Hwang YK, Tan JC (2016). Freestanding fiber mats of zeolitic imidazolate framework 7 via one-step, scalable electrospinning. J. Appl. Polym. Sci..

[6] Balsamo M, Silvestre-Albero A, Silvestre-Albero J, Erto A, Rodriguez-Reinoso F, Lancia A (2014). Assessment of CO2 adsorption capacity on activated carbons by a combination of batch and dynamic tests. Langmuir.

[7] Wjihi S, Aouaini F, Erto A, Balsamo M, Lamine AB (2021). Advanced interpretation of CO2 adsorption thermodynamics onto porous solids by statistical physics formalism. Chem. Eng. J..

[8] Abbasi A, Nasef MM, Faridi-Majidi R, Etesami M, Takeshi M, Abouzari-Lotf E (2018). Highly flexible method for fabrication of poly (Glycidyl Methacrylate) grafted polyolefin nanofiber. Radiat. Phys. Chem..

[9] Zhuang L, Chen S, Lin R, Xu X (2013). Preparation of a solid amine adsorbent based on polypropylene fiber and its performance for CO2 capture. J. Mater. Res..

[10] Ashrafi F, Firouzzare M, Javad Ahmadi S, Reza Sohrabi M, Khosravi M (2019). Preparation and modification of forcespun polypropylene nanofibers for adsorption of uranium (VI) from simulated seawater. Ecotoxicol. Environ. Saf..

[11] Tajer M, Anbia M, Salehi S (2021). Fabrication of polyacrylonitrile hybrid nanofiber scaffold containing activated carbon by electrospinning process as nanofilter media for SO2, CO2, and CH4 adsorption. Environ. Prog. Sustain. Energy.

[12] Bhardwaj N, Kundu SC (2010). Electrospinning: A fascinating fiber fabrication technique. Biotechnol. Adv..

[13] Abbasi A, Nasef MM, Takeshi M, Faridi-Majidi R (2014). Electrospinning of nylon-6, 6 solutions into nanofibers: Rheology and morphology relationships. Chin. J. Polym. Sci..

[14] Zainab G, Iqbal N, Babar AA, Huang C, Wang X, Yu J, Ding B (2017). Free-standing, spider-web-like polyamide/carbon nanotube composite nanofibrous membrane impregnated with polyethyleneimine for CO2 capture. Compos. Commun..

[15] Olivieri L, Roso M, De Angelis MG, Lorenzetti A (2018). Evaluation of electrospun nanofibrous mats as materials for CO2 capture: A feasibility study on functionalized poly (acrylonitrile)(PAN). J. Membr. Sci..

[16] Nasef MM, Hegazy E-SA (2004). Preparation and applications of ion exchange membranes by radiation-induced graft copolymerization of polar monomers onto non-polar films. Prog. Polym. Sci..

[17] Nasef MM, Güven O (2012). Radiation-grafted copolymers for separation and purification purposes: Status, challenges and future directions. Prog. Polym. Sci..

[18] Rojek T, Gubler L, Nasef M, Abouzari-Lotf E (2017). Polyvinylamine-containing adsorbent by radiation-induced grafting of N-vinylformamide onto ultrahigh molecular weight polyethylene films and hydrolysis for CO2 capture. Ind. Eng. Chem. Res..

[19] Abbasi A, Nasef MM, Kheawhom S, Faridi-Majidi R, Takeshi M, Abouzari-Lotf E, Choong T (2019). Amine functionalized radiation induced grafted polyolefin nanofibers for CO2 adsorption. Radiat. Phys. Chem..

[20] Torkaman R, Maleki F, Gholami M, Torab-Mostaedi M, Asadollahzadeh M (2021). Assessing the radiation-induced graft polymeric adsorbents with emphasis on heavy metals removing: A systematic literature review. J. Water Process Eng..

[21] Maleki F, Gholami M, Torkaman R, Torab-Mostaedi M, Asadollahzadeh M (2022). Influence of phosphonic acid as a functional group on the adsorption behavior of radiation grafted polypropylene fabrics for Co(II) removal. Radiat. Phys. Chem..

[22] Imanian Z, Hormozi F, Torab-Mostaedi M, Asadollahzadeh M (2022). Highly selective adsorbent by gamma radiation-induced grafting of glycidyl methacrylate on polyacrylonitrile/polyurethane nanofiber: Evaluation of CO2 capture. Sep. Purif. Technol..

[23] Madani M, El-Sayed S (2007). Radiation effects on optical properties of ethylene vinyl acetate copolymer films. J. Macromol. Sci. Part B Phys..

[24] Maleki F, Torkaman R, Torab-Mostaedi M, Asadollahzadeh M (2022). Optimization of grafted fibrous polymer preparation procedure as a new solid basic catalyst for biodiesel fuel production from palm oil. Fuel.

[25] Lubna MM, Salem KS, Sarker M, Khan MA (2018). Modification of thermo-mechanical properties of recycled PET by vinyl acetate (VAc) monomer grafting using gamma irradiation. J. Polym. Environ..

[26] Shamsaei E, Nasef M, Saidi H, Yahaya A (2014). Parametric investigations on proton conducting membrane by radiation induced grafting of 4-vinylpyridine onto poly (vinylidene fluoride) and phosphoric acid doping. Radiochim. Acta.

[27] Maleki F, Gholami M, Torkaman R, Torab-Mostaedi M, Asadollahzadeh M (2021). Multivariate optimization of removing of cobalt(II) with an efficient aminated-GMA polypropylene adsorbent by induced-grafted polymerization under simultaneous gamma-ray irradiation. Sci. Rep..

[28] Maleki F, Gholami M, Torkaman R, Torab-Mostaedi M, Asadollahzadeh M (2021). Cobalt(II) removal from aqueous solution by modified polymeric adsorbents prepared with induced-graft polymerization: Batch and continuous column study with analysis of breakthrough behaviors. Environ. Technol. Innov..

[29] Yang Y, Li H, Chen S, Zhao Y, Li Q (2010). Preparation and characterization of a solid amine adsorbent for capturing CO2 by grafting allylamine onto PAN fiber. Langmuir.

[30] Zubair NA, Nasef MM, Mohamad NA, Abouzari-Lotf E, Ting TM, Abdullah EC (2020). Kinetic studies of radiation induced grafting of N-vinylformamide onto polyethylene/polypropylene fibrous sheets and testing its hydrolysed copolymer for CO2 adsorption. Radiat. Phys. Chem..

[31] Choi SH, Lee KP, Nho YC (2001). Adsorption of urokinase by polypropylene films with various amine groups. J. Appl. Polym. Sci..

[32] Serna-Guerrero R, Sayari A (2010). Modeling adsorption of CO2 on amine-functionalized mesoporous silica: 2: Kinetics and breakthrough curves. Chem. Eng. J..

[33] Chen C, Chen Z, Shen J, Kang J, Zhao S, Wang B, Chen Q, Li X (2021). Dynamic adsorption models and artificial neural network prediction of mercury adsorption by a dendrimer-grafted polyacrylonitrile fiber in fixed-bed column. J. Clean. Prod..

[34] Kumari U, Mishra A, Siddiqi H, Meikap B (2021). Effective defluoridation of industrial wastewater by using acid modified alumina in fixed-bed adsorption column: Experimental and breakthrough curves analysis. J. Clean. Prod..

[35] Chu KH (2020). Breakthrough curve analysis by simplistic models of fixed bed adsorption: In defense of the century-old Bohart-Adams model. Chem. Eng. J..

[36] Patel H (2019). Fixed-bed column adsorption study: A comprehensive review. Appl Water Sci.

[37] Dovi E, Aryee AA, Kani AN, Mpatani FM, Li J, Li Z, Qu L, Han R (2021). Functionalization of walnut shell by grafting amine groups to enhance the adsorption of Congo red from water in batch and fixed-bed column modes. J. Environ. Chem. Eng..

[38] Demarchi CA, Debrassi A, Dal Magro J, Nedelko N, Ślawska-Waniewska A, Dłużewski P, Greneche J-M, Rodrigues CA (2015). Adsorption of Cr (VI) on crosslinked chitosan–Fe (III) complex in fixed-bed systems. J. Water Process Eng..

[39] Cruz MAP, Guimarães LCM, da Costa Júnior EF, Rocha SDF, Mesquita PDL (2020). Adsorption of crystal violet from aqueous solution in continuous flow system using bone char. Chem. Eng. Commun..

[40] Wang Y, Chen Y, Wang C, Sun J, Zhao Z, Liu W (2018). Polyethylenimine-modified membranes for CO2 capture and in situ hydrogenation. ACS Appl. Mater. Interfaces..

[41] Nasef MM, Abbasi A, Ting T (2014). New CO2 adsorbent containing aminated poly (glycidyl methacrylate) grafted onto irradiated PE-PP nonwoven sheet. Radiat. Phys. Chem..

